# Sediment microbial community structure associated to different ecological types of mangroves in Celestún, a coastal lagoon in the Yucatan Peninsula, Mexico

**DOI:** 10.7717/peerj.14587

**Published:** 2023-02-08

**Authors:** Elizabeth Selene Gómez-Acata, Claudia Teutli, Luisa I. Falcón, José Q. García-Maldonado, Alejandra Prieto-Davó, Alfredo Yanez-Montalvo, Santiago Cadena, Xavier Chiappa-Carrara, Jorge A. Herrera-Silveira

**Affiliations:** 1 Instituto de Ecología, UNAM, Mérida, Yucatán, México; 2 Escuela Nacional de Estudios Superiores, Mérida, Yucatán, México; 3 Laboratorio Nacional de Resiliencia Costera (LANRESC), Sisal, Yucatán, México; 4 Centro de Investigación y de Estudios Avanzados del Instituto Politécnico Nacional, Mérida, Yucatán, México; 5 Unidad de Química en Sisal, Facultad de Química, UNAM, Sisal, Yucatán, México; 6 Unidad Multidisciplinaria de Docencia e Investigación, Unidad Sisal, Universidad Nacional Autónoma de México, Sisal, Yucatán, México

**Keywords:** Mangrove microbiome, Basin mangrove, Dwarf mangrove, Fringe mangrove, *Desulfatiglicans*, *Avicennia germinans*, *Rhizophora mangle*

## Abstract

Mangroves are unique coastal ecosystems, which have many important ecological functions, as they are a reservoir of many marine species well adapted to saline conditions and are fundamental as sites of carbon storage. Although the microbial contribution to nutrient cycling in these ecosystems has been well recognized, there is a lack of information regarding the microbial composition and structure of different ecological types of mangrove forests. In this study, we characterized the microbial community (Bacteria and Archaea) in sediments associated with five ecological types of mangrove forests in a coastal lagoon dominated by *Avicennia germinans* and* Rhizophora mangle*, through 16S rRNA-V4 gene sequencing. Overall, Proteobacteria (51%), Chloroflexi (12%), Gemmatimonadetes (5%) and Planctomycetes (6%) were the most abundant bacterial phyla, while Thaumarchaeota (30%), Bathyarchaeota (21%) and Nanoarchaeaeota (18%) were the dominant archaeal phyla. The microbial composition associated with basin mangroves dominated by *Avicennia germinans* was significantly different from the other ecological types, which becomes relevant for restoration strategies.

## Introduction

Mangrove ecosystems cover more than 200,000 km^2^ of tropical and subtropical coastlines (Duke et al., 2007). They are considered among the most valuable coastal ecosystems, since they provide numerous ecosystem services including protection against typhoons/hurricanes, tsunamis and floods, store nutrients and provide shelter for many marine species (Walters et al., 2008; Polidoro et al., 2010; Alongi, 2011). They contribute between 10 to 15% of carbon storage in coastal sediments and export 10 to 11% of the terrestrial carbon in particles to the ocean (Alongi, 2014). It has been estimated that the carbon biomass in mangrove sediments is approximately three times the biomass that constitutes the mangrove vegetation (Hossain & Nuruddin, 2016). These ecosystems have a relative structural simplicity, due to the few tree species present. They are adapted to the intertidal zone, so they develop in saline, flooded anoxic soils. Mangroves are facultative halophytes and reach their maximum development in estuarine conditions resulting in a typological variability of mangrove ecosystems, which are an expression of environmental conditions. Climate, geomorphology, regulatory stressors (salinity, temperature, pH, redox potential) and hydroperiod (Twilley & Rivera-Monroy, 2005) are factors that contribute to the different ecological types of mangroves: fringe, basin, dwarf, riverine and hammock or peten, which is characteristic of the Yucatan Peninsula (YP).

In particular, the YP presents a variability of coastal communities that reflect the geological characteristics of the region, this being an oligotrophic karst configuration (Herrera-Silveira & Morales-Ojeda, 2009), with a highly permeable carbonate substrate type and a system subsurface hydrology with dominant flows to the eastern and western extremes of the area known as the “cenote ring” (Perry et al., 1995; Bauer-Gottwein et al., 2011). The geohydrology of the YP coast determines the development of mangrove scenarios that can be wet or dry (Zaldívar-Jiménez et al., 2010). The wet scenario is characterized by the function of the cenote (sinkhole) ring, which concentrates and directs groundwater to sites of interception with the coast. The dry scenario is characterized by flooding and water contributions strongly influenced by the seasonality of precipitation and less presence of freshwater from sinkholes and fractures favoring sediments with hypersaline conditions and low nutrient concentration.

Within the mangrove ecosystems, microbes are critical components, occupying a wide variety of niches which are essential for carbon, nitrogen and phosphorus accumulation, transformation and fate as part of the ecological role of the mangroves as sink, source and transformation of these essential elements (Raynaud & Nunan, 2014). However, the ecological role of the microbial components of mangrove ecosystems is still poorly understood. The microbial community in mangrove sediments is dynamic and diverse, with bacteria and fungi comprising most of the total biomass, followed by algae and protozoa (Sahoo & Dhal, 2009). Yet, there is a lack of knowledge regarding the composition of the microbial communities in different ecological types of mangroves, specifically in karstic environments. Hence, understanding if there are patterns in microbial composition specific to different mangrove types, and if there is a common core community among mangroves, becomes essential to unravel the ecology of these environments. Hence, the aim of this study was to determine the structure and composition of sediment microbial communities in different ecological types of mangroves from Celestún, a coastal lagoon located in the karstic scenario of the Yucatan Peninsula, including fringe mangroves dominated by *R. mangle* (FRm), basin mangroves dominated by *R. mangle* and *A. germinans* (BRmAg), basin mangroves dominated by *R. mangle* (BRm), basin mangroves dominated by *A. germinans* (BAg) and a dwarf mangrove dominated by *R. mangle* (DRm).

## Material and Methods

### Study site

**Figure 1:**
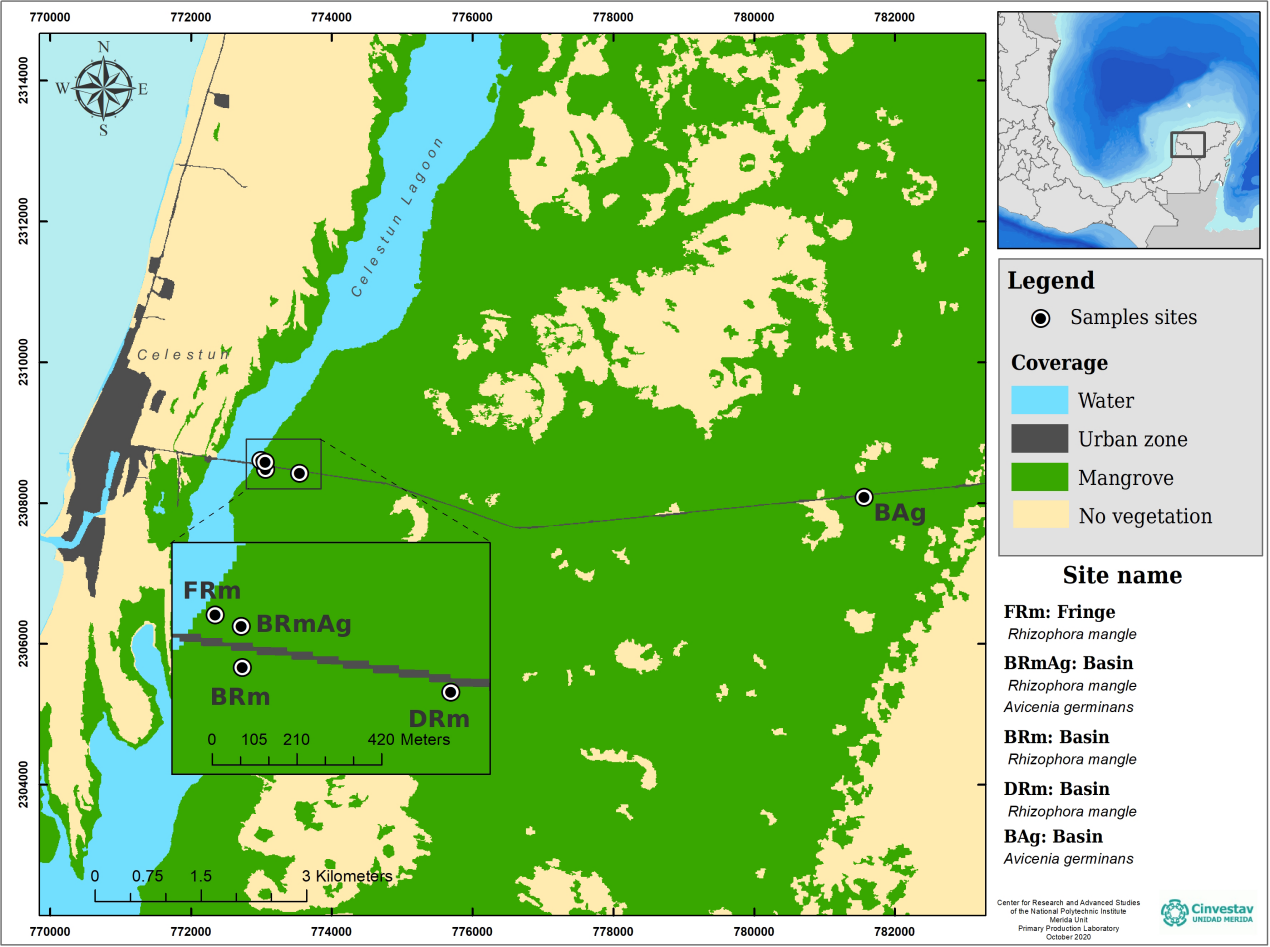
Study sites and ecological mangrove forests in Celestún.

This study was carried out in the central region of Celestún Lagoon, located in the northwest corner of the YP (Fig. 1). The lagoon has an extension of 23 km and a maximum width of 2 km; the depth varies between 0.5–2.5 m, covering an area of approximately 28 km^2^ (Herrera-Silveira, Ramírez & Zaldívar, 1998). It has a mouth of approximately 0.46 km located in the south, which is its only communication with the sea. The dominant vegetation is constituted by mangrove forests, which due to the shallow depth of the aquifer, as well as a scarce elevation and low topography, favors the development of different ecological types of mangroves (Table 1). Celestún is a tropical coastal lagoon with a semi-diurnal tidal regime (Vega-Cendejas, Hernández & Cruz, 1997), warm weather (average of 28.5 °C) (García & Mosiño, 1992), that has three main seasons, including a dry spring, rainy summer and cool winter (Herrera-Silveira, 1994; Herrera-Silveira, Ramírez & Zaldívar, 1998; Teutli-Hernández et al., 2019).

**Table 1:** Location of ecological mangrove types and structural characteristics.

Mangrove type	Sample code	Coordinates	Dominant species	Diameter (cm)	Height (m)
Basin	BAg	20.851527 N – 90.294222 W	*A. germinans*	14.3 ± 3	5 ± 1
Dwarf	DRm	20.855805 N – 90.371222 W	*R. mangle*	2.0 ± 0	2 ± 0
Basin	BRm	20.856361 N – 90.375861 W	*R. mangle*	9.0 ± 1	5 ± 0
Basin	BRmAg	20.857277 N – 90.375888 W	*R. mangle* and *A. germinans*	26.0 ± 6	10 ± 3
Fringe	FRm	20.857527 N – 90.376472 W	*R. mangle*	28.0 ± 9	14 ± 2

### Sediment sampling

In each mangrove type, two sediment cores were collected for biogeochemical characterization using a 1 m metal corer with a diameter of 5.25 cm. Surface sediments (first 2 cm/1gr), associated to mangrove root development, were collected in three sites per mangrove type in replicates (*n* = 4) (N =12/per mangrove type) for molecular analysis. Sediments were immediately stored in cryogenic tubes, frozen in liquid nitrogen and transported to the lab for storage at −80 °C.

Bulk density, organic matter and nutrient contents (total carbon, total nitrogen and total phosphorus) were measured for each sample. To determine the apparent density, the first 15 cm of the sediment were analyzed, dried in an oven at 60 °C and the bulk density was calculated based on the ratio between the dry weight and volume. The organic matter content of the sediment was determined gravimetrically after each portion was burned to ash in a muffle furnace at 550 °C for 4 h (Chen & Twilley, 1999). To determine total carbon and nitrogen, the samples were ground and homogenized; 20–30 mg were subsequently weighed in tin capsules in triplicates and these were analyzed with an automatic elemental analyzer, model FLASH-EA-1112 (Quest). Total phosphorus was measured by colorimetry following the methodology described by Aspila, Agemian & Chau (1976) and Parsons, Maita & Lalli (1984). In each of the plots, three pore-water samples were taken at 40 cm depth using an acrylic tube and syringe. A refractometer (Atago, Tokyo, Japan) was used to measure salinity and a ULTRAMETER II-6 was used to measure temperature, pH and redox potential (Tables 2 and 3).

### DNA extraction and 16SrRNA gene amplification

The microbial community composition of each ecological mangrove type was estimated with high-throughput sequencing of the 16SrRNA-V4 region. Total sediment DNA extraction was performed with the DNeasy PowerSoil Kit (Qiagen, Hilden, Germany) with 0.25 g following the manufacturer’s protocol. Extracted DNA was stored at −70 °C in the laboratory until PCR amplification. The V4 hypervariable region of the 16SrRNA gene was amplified using primers 515F/806R (Caporaso et al., 2011). Each PCR reaction contained 1 µl of DNA, 2.5 µl Takara ExTaq PCR buffer 10X, 2 µl Takara dNTP mix (2.5 mM), 0.7 µl BSA (20 mg ml^−1^), 1 µl each primer (10 µM), 0.125 µl Takara Ex Taq DNA Polymerase (5 U µl^−1^) (TaKaRa, Shiga, Japan) and nuclease free-water to a final volume of 25 µl. PCR was performed in triplicates using the following conditions: 95 °C for 3 min, followed by 35 cycles at 95 °C for 30 s, 52 °C for 40 s and 72 °C for 90 s, and a final extension step at 72 °C for 12 min. Amplicon triplicates were pooled and cleaned with Ampliclean carboxyl-coated magnetic beads (NimaGen, NDL), then quantified using a Qubit instrument with the Qubit High Sensitivity DNA kit (Invitrogen, Carlsbad, CA). The purified 16SrRNA amplicons were pooled in equal amounts (20 ng µl^−1^) and sequenced with an Illumina MiSeq (Illumina, San Diego, CA, USA). Sequence reads can be found in the NCBI Sequence Read Archive under Bioproject number PRJNA550111, SRA accession numbers SRR9973295–SRR9973347.

**Table 2:** Interstitial and superficial water physicochemical characteristics measured *in situ* for different mangrove types (mean ± SD).

	Interstitial water				Superficial water			
Mangrove type	Salinity (psu)	Temperature (°C)	pH	Redox potential (mv)	Salinity (psu)	Temperature (°C)	pH	Redox potential (mv)
BAg	34.7 ± 11.1	28.4 ± 0.3	7.0 ± 0.6	−219.2 ± 41.7	4.0 ± 1.7	28.0 ± 0.3	7.6 ± 0.0	−223.6 ± 18.8
DRm	54.2 ± 0.1	30.8 ± 0.9	6.7 ± 0.0	−239.5 ± 0.1	3.3 ± 0.6	28.8 ± 0.1	7.5 ± 0.3	−187.3 ± 47.9
BRm	49.7 ± 3.4	28.6 ± 0.4	6.7 ± 0.1	−254.0 ± 3.4	5.0 ± 0.0	27.3 ± 0.9	7.3 ± 0.4	−224.0 ± 20.7
BRmAg	49.7 ± 1.3	28.0 ± 0.2	6.5 ± 0.0	−239.0 ± 12.2	11.3 ± 4.0	28.4 ± 0.4	7.8 ± 0.0	−239.3 ± 18.2
FRm	30.2 ± 1.7	26.7 ± 0.1	6.7 ± 0.0	−245.3 ± 5.6	10.3 ± 2.5	27.7 ± 1.3	7.2 ± 0.5	−247.3 ± 15.2

**Table 3:** Physicochemical characteristics of sediments in different mangrove types (mean ± SD).

Mangrove type	Total carbon (%)	Organic matter (%)	Total nitrogen (%)	Total phosphorus (%)	Density (g/cm^3^)
BAg	63.20 ± 13.40	26.52 ± 0.68	2.11 ± 0.37	0.03 ± 0.04	0.27 ± 0.06
DRm	43.30 ± 3.30	21.61 ± 2.80	1.10 ± 0.20	0.06 ± 0.01	0.16 ± 0.07
BRm	61.15 ± 4.26	30.46 ± 1.01	2.02 ± 0.17	0.06 ± 0.01	0.18 ± 0.02
BRmAg	62.64 ± 4.26	27.58 ± 0.81	1.91 ± 0.29	0.02 ± 0.03	0.20 ± 0.05
FRm	55.07 ± 5.60	30.57 ± 7.67	1.59 ± 0.45	0.04 ± 0.02	0.28 ± 0.05

### Data analysis

Sequences were analyzed with QIIME 2 2018.6 (Bolyen et al., 2018). Paired-end sequences were demultiplexed and quality filtered with q2-plugin *demux emp-paired*. Sequences with less than 10 of quality score were discarded; trimming was done at position 14 in both forward and reverse reads. Chimera and singletons were removed, and denoising was done using the plugin *qiime dada2 denoise-paired* with DADA2 (Callahan et al., 2016): Sequences were merged with a 200 bp threshold and clustered into amplicon sequence variants (ASVs). Taxonomy was assigned to ASVs using qiime plugin *feature-classifier classify-consensus-vsearch* with VSEARCH (Rognes et al., 2016) against the SILVA database (release 132–99% OTUs, 515-806 region, L7 taxonomy). The class Bathyarchaeia (Archaea) from SILVA database was named as phylum Bathyarchaeota in the figures, since it was assigned to a novel phylum (Meng et al., 2014). Mitochondrial and chloroplast sequences were filtered from the feature table and the representative sequences file and ASVs present in less than two samples were filtered from both the feature table and the sequences file. Archaeal sequences were filtered in an independent feature table and sequence file, and analyzed independently from Bacteria. Bacteria and Archaea feature tables were rarified to 13,917 and 1,021 ASVs per sample respectively (Fig. S1). Alignment was done with mafft (Katoh et al., 2002) and used to construct a phylogeny with FastTree2 (Price, Dehal & Arkin, 2010).

The alpha diversity (diversity within the samples) was calculated using three different metrics: Shannon, Simpson and observed ASVs with the plugin *qiime diversity alpha*. Phylogenetic beta diversity analysis was measured using weighted Unifrac (Lozupone et al., 2007) and unweighted Unifrac (Lozupone & Knight, 2005) phylogenetic distance metrics and visualized with a principal coordinate analysis (PCoA) done with the plugin *qiime diversity core-metrics-phylogenetic*, the 3D PCoA plot was visualized using emperor (Vazquez-Baeza et al., 2013). In order to detect significant differences in alpha diversity between the ecological types of mangroves, a Kruskal-Wallis test was performed in R (v 4.0.3) (R Core Team, 2020). For beta diversity, a PERMANOVA test was done with the Adonis function from vegan (v2.5-7) (Oksanen et al., 2020) using the weighted Unifrac distance matrix with 999 permutations.

### Indicator taxa analysis

ITA was performed with the rarefied Bacterial and Archaeal feature table. The indicator species value (IndVal) (Dufrêne & Legendre, 1997) was calculated in the *indicspecies* package ver 1.7.1 (Cáceres & Legendre, 2009) from R (R Core Team, 2020). The IndVal value was used to identify specific ASV’s for each ecological mangrove type and those shared between mangroves. In this study, we considered as indicator species those with IndVal values > 0.95 and *P* > 0.001.

## Results

In an effort to understand the microbial community in sediments associated with roots of different ecological types of mangroves, we studied the changes in their diversity across a transect from near-shore to inland in Celestún Lagoon, YP.

### Physicochemical characteristics

Interstitial water in the different ecological types of mangroves ranged between 54.2 psu (DRm) and 30.2 psu (FRm), with temperatures ranging between 30.8 °C (DRm) and 26.7 °C (FRm). All mangrove types had similar pH (6.5–7) and Redox potential (−254 and −219.2 mv) (Table 2). Superficial water salinity ranged between 11.3 psu (BRmAg) and 3.3 psu (DRm), and the BAg mangrove, which is the most inland point in this study, had a salinity of 4 psu (Table 2). Superficial water temperature and pH were similar for all study sites (27.3–28.8 °C, 7.2−7.8) and Redox potential ranged from −187.3–247.3 mv (Table 2).

Regarding mangrove sediments, the DRm mangrove type recorded the lowest values of organic matter (21.61%) and apparent density (0.16 g/cm^3^), as well as the lowest total carbon (43.3%) and total nitrogen content (1.10%), while the highest contents of total carbon and nitrogen content were found in BAg (63.20%, 2.11%). The highest apparent density (0.28 g/cm^3^) and organic matter (30.57%) were recorded in the FRm mangrove. Total phosphorus values were low, with highest values recorded in the DRm mangrove (0.06%). There were no significant differences between physicochemical characteristics analyzed in sediment samples collected for this study (Kruskal-Wallis test).

### Bacterial structure and composition

A total of 4,276,500 raw sequence reads for the V4 region of the 16SrRNA gene were obtained after quality filtering. Removal of mitochondrial and chloroplast sequences left 4,272,606 ASVs for posterior analysis. A total of 11,503 different ASVs for Bacteria in 53 samples along the transect were obtained. Alpha diversity index showed that BAg had the lowest microbial diversity of all mangrove-types analyzed. The Shannon estimator values ranged from 8.0 to 9.6; Simpson index ranged from 0.9 to 1.0 and Observed ASVs ranged from 597.2 to 1597.6. Significant differences in alpha diversity between mangrove types are shown using the Krustal-Wallis test (Table S1). The rarefaction curves show enough sequencing depth to represent bacterial diversity (Fig. S1). A total of 58 phyla, 171 classes, 416 orders, 656 families and 965 genera were recovered.

**Figure 2:**
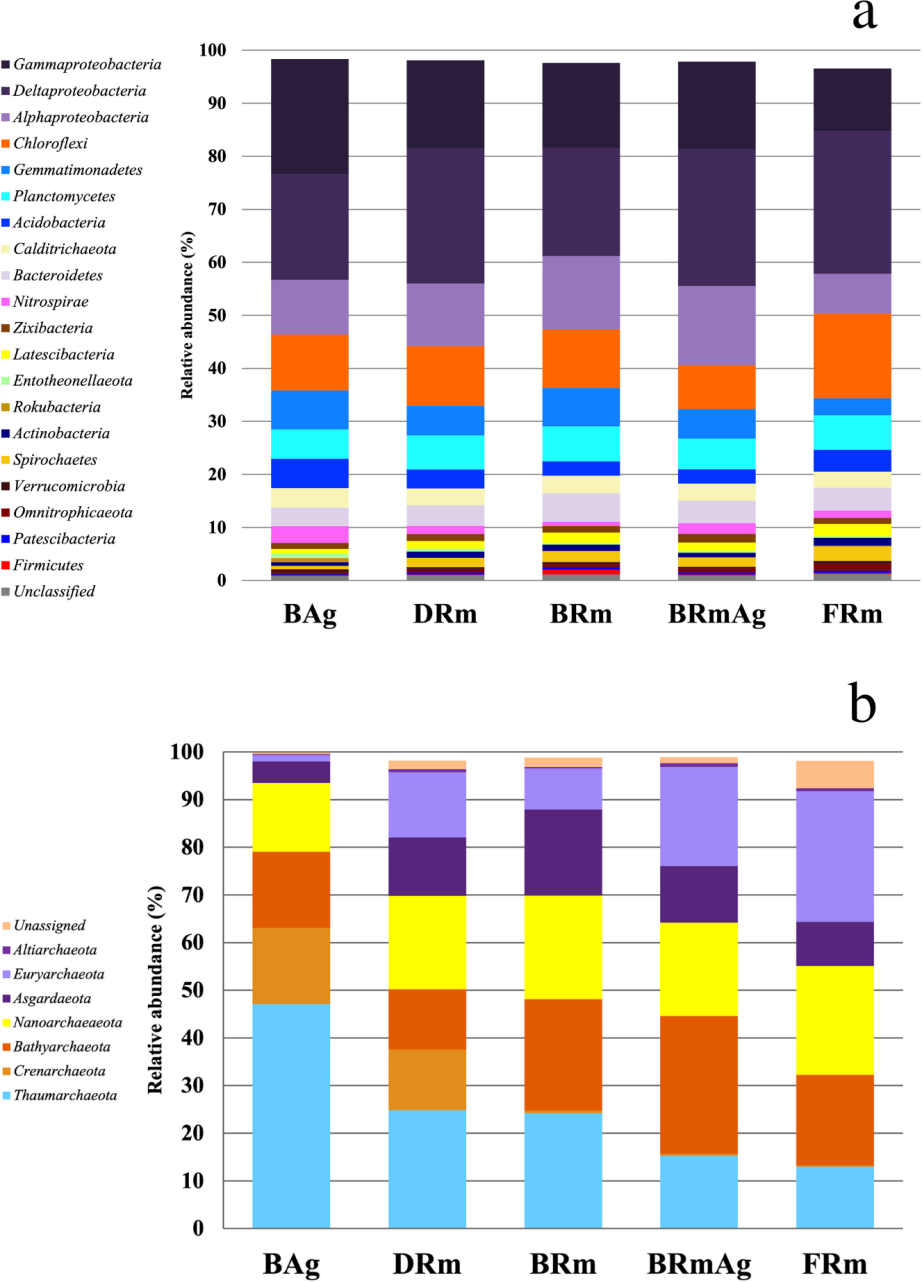
Relative abundance of the microbial communities of sediments associated with five different mangrove forests from Celestún Lagoon. (A) Bacterial phyla (Archaeal phyla).

The most abundant bacterial phyla in all samples were Proteobacteria (46% to 57%), followed by Chloroflexi (8% to 16%), Gemmatimonadetes (3% to 7%) and Planctomycetes (6% to 7%) (Fig. 2A). However, there was a significant difference in the relative abundance of these phyla between mangroves according to a Kruskal-Wallis test (*p* < 0.001). Moreover, *post hoc* Dunn test showed that BAg and FRm were significantly different (*p* < 0.001) in the abundance of Gemmatimonadetes, Acidobacteria, Nitrospirae and Firmicutes (Table S2). The most abundant classes in all mangroves were Deltaproteobacteria (20% to 27%), Gammaproteobacteria (12% to 22%), Alphaproteobacteria (8% to 15%) and Anaerolineae (5% to 10%). The most abundant families were Desulfobacteraceae (4% to 8%), Nitrosococcaceae (2% to 7%) and Desulfarculaceae (2% to 6%) (Fig. 2B). Moreover, Sva0081 sediment group (3% to 5%), AqS1 (1% to 5%) and *Desulfatiglans* (2% to 6%) were the most abundant genera (Fig. S2b). The most abundant class in DRm, BRm, BRmAg and FRm was Deltaproteobacteria and in BAg, Gammaproteobacteria. The order MBMPE27 accounted for 8% in BAg while in the rest of mangroves it was less than 1% (Fig. S2B). The most abundant family in BAg, DRm, BRmAg and FRm was Desulfobacteriaceae while in BRm was Desulfarculaceae (Fig. S2A).

**Figure 3:**
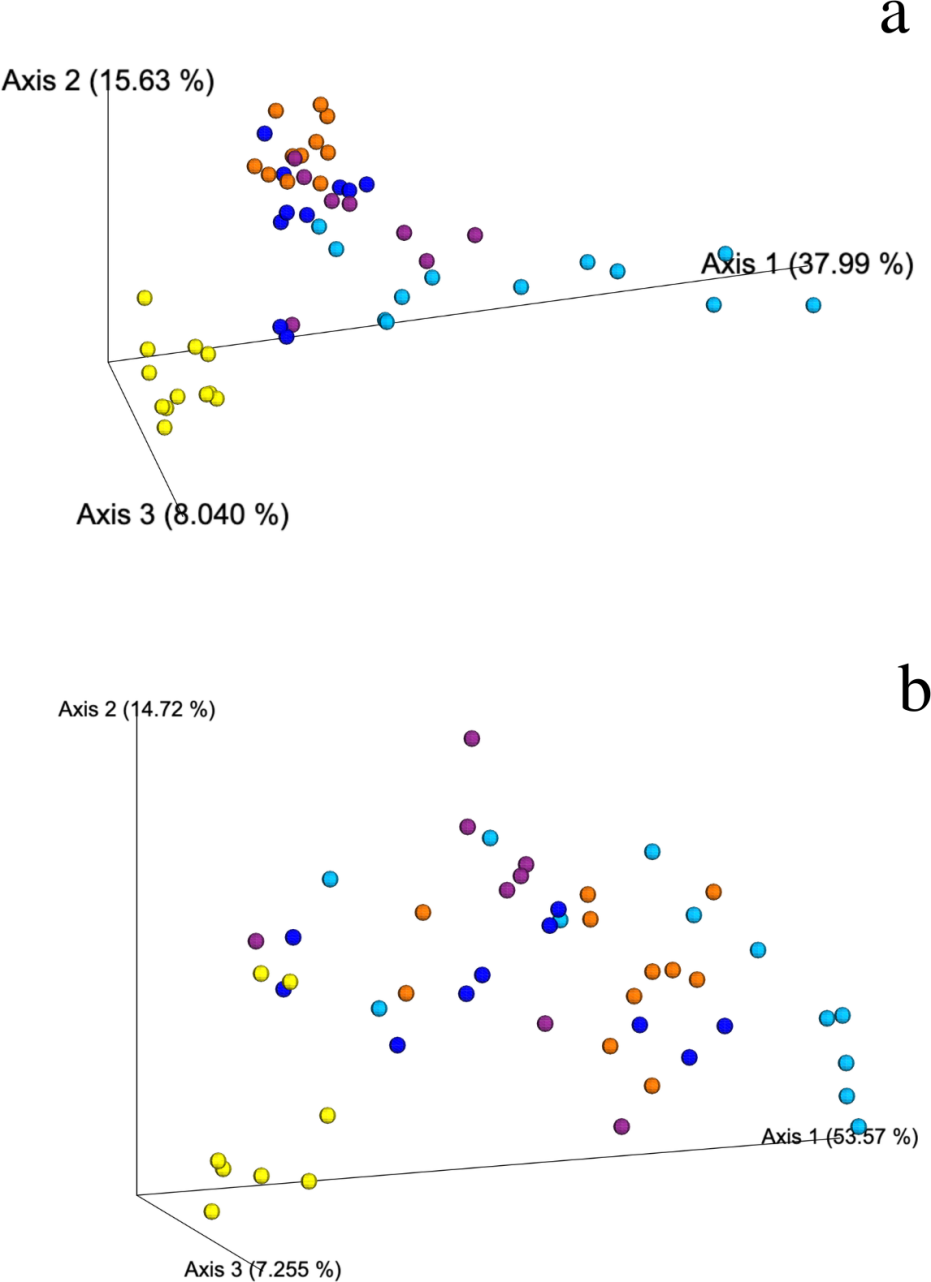
Weighted Unifrac PCoA showing the spatial ordination of ASVs of different ecological types of mangrove forests in Celestún. (A) Bacteria and (B) Archaea BAg (yellow), DRm (blue), BRm (purple), BRmAg (orange), FRm (cyan).

Weighted and unweighted Unifrac analyses show that DRm, BRm and BRmAg mangrove sediments grouped together suggesting a similar bacterial composition, which is significantly different from that found in BAg mangrove sediment samples (PERMANOVA, *R*^2^ = 0.468, *p* = 0.001); FRm had the least clustered composition, sharing bacterial ASVs with the rest of the *R. mangle* ecological types, yet showing a unique composition (Fig. 3A; Fig. S3). In addition, no physicochemical variables included in this study were significantly associated with the changes observed in the microbial community (Table S3), suggesting differences in microbial composition are associated with mangrove rhizobiome.

### Bacterial indicator taxa

**Figure 4:**
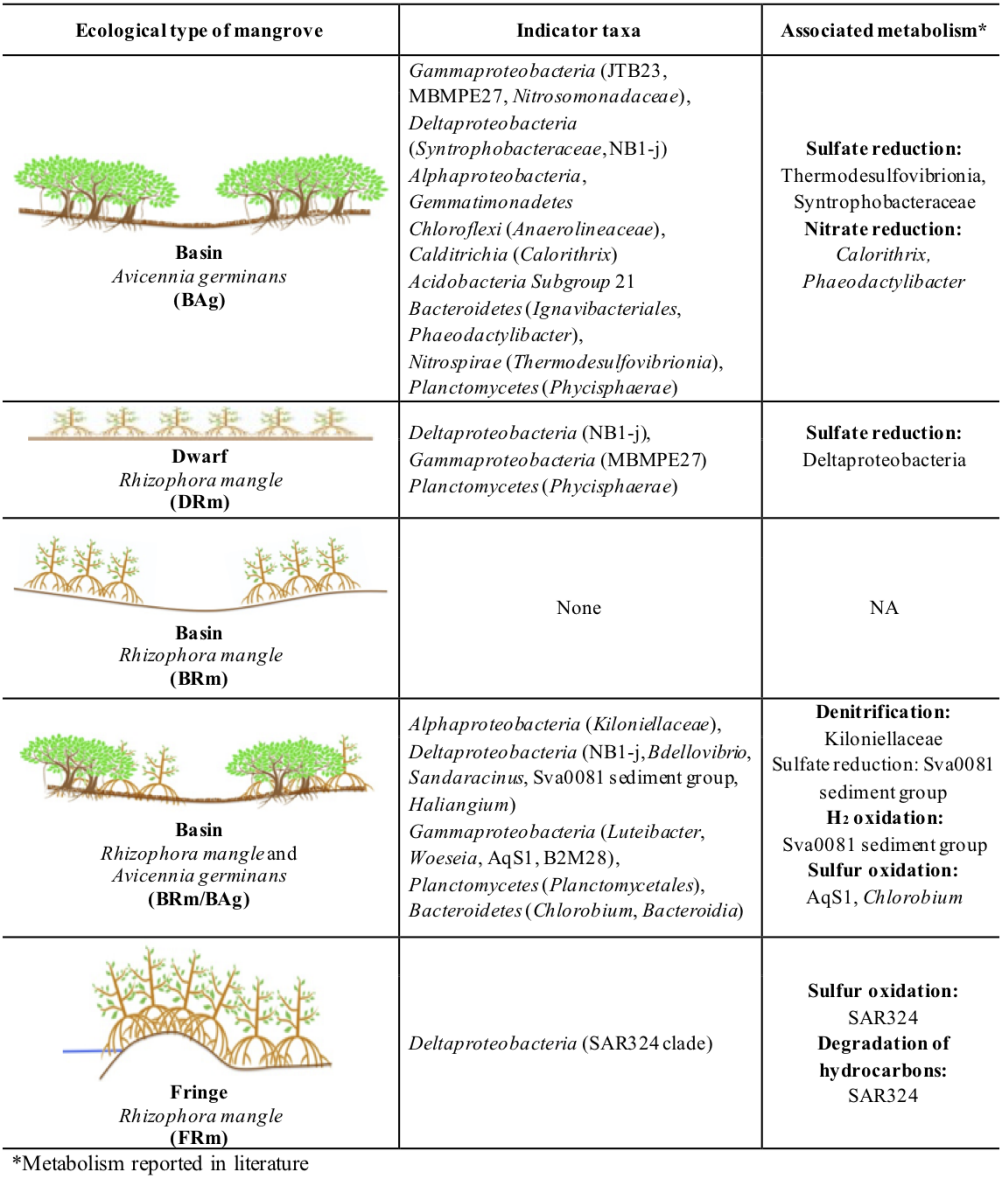
Bacterial Indicator Taxa for each ecological type of mangrove forests from Celestún.

**Figure 5:**
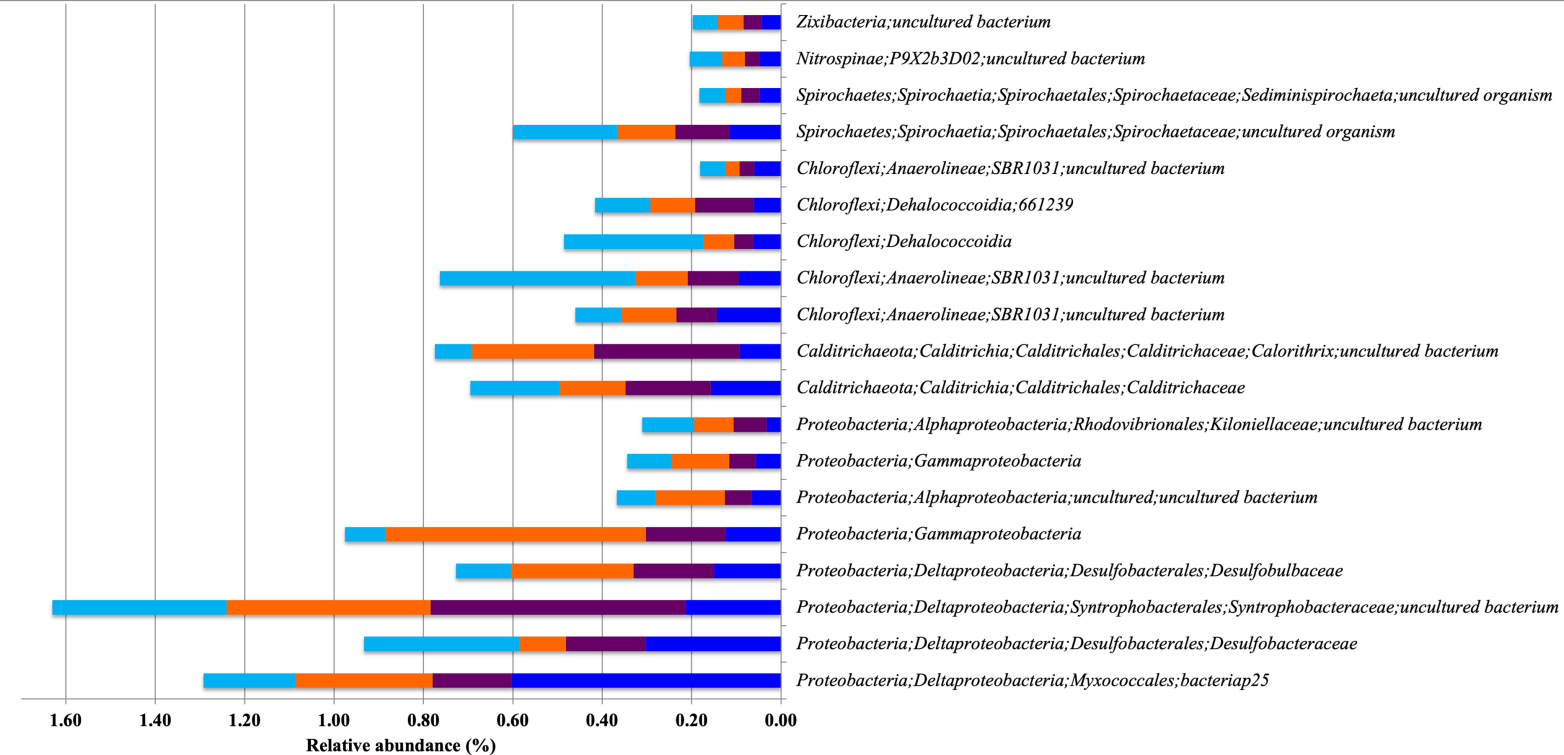
Relative abundance of Bacterial Indicator Taxa that are shared between different types of mangrove forests. DRm (blue), BRm (purple), BRmAg (orange), FRm (cyan).

Indicator Taxa Analysis (ITA) for bacteria shows that BAg had 20 indicator taxa, while DRm had five, BRmAg had 16 and FRm only two; BRm did not have any indicator taxa. The indicator taxa in BAg belong to eight different phyla: Proteobacteria (class Gammaproteobacteria, Deltaproteobacteria and Alphaproteobacteria), Gemmatimonadetes (class Gemmatimonadetes), Chloroflexi (class Anaerolineae), Acidobacteria (class Subgroup 21), Calditrichaeota (class Calditrichia), Bacteroidetes (class Ignavibacteria and Bacteroidia), Nitrospirae (class Thermodesulfovibrionia) and Planctomycetes (class Phycisphaerae). The DRm has five indicator taxa belonging to phyla Proteobacteria (class Gammaproteobacteria and Deltaproteobacteria) and Planctomycetes (class Phycisphaerae); in the BRmAg indicator taxa belong to three phyla: Proteobacteria (class Gammaproteobacteria, Deltaproteobacteria and Alphaproteobacteria), Planctomycetes (class Planctomycetacia) and Bacteroidetes (class Chlorobia and Bacteroidia); in FRm indicator taxa belong only to Proteobacteria (class Deltaproteobacteria). The most abundant bacterial indicator taxa was MBMPE27 (Phylum Proteobacteria, class Gammaproteobacteria) (9.01%) in BAg mangrove (Fig. 4; Fig. S4; Table S4). There were 19 indicator taxa shared between DRm, BRm, BRmAg and FRm, and absent from BAg, that belong to six different phyla: Proteobacteria, Calditrichaeota, Chloroflexi, Spirochaetes, Nitrospinae and Zixibacteria (Fig. 5).

### Archaeal composition

A total of nine phyla, 22 classes, 53 orders, 14 families and 71 archaeal genera were recovered in this study. Rarefaction curves show enough sequencing depth to get a complete representation of the archaeal diversity in the mangrove sediments from this study (Fig. S5). BAg showed the lowest diversity among all mangroves (Table S1). The most abundant phyla in all mangroves were Thaumarchaeota (13% to 47%), followed by Bathyarchaeota (13% to 29%) and Nanoarchaeaeota (14% to 23%) (Fig. 2B). The Kruskal-Wallis test showed that the abundance of Crenarchaeota, Thaumarchaeota and Euryarchaeota were different between BAg and FRm (*p* < 0.001) (Table S2). The most abundant classes were Nitrososphaeria (12% to 24%), Woesearchaeia (7% to 18%) and Thermoplasmata (1% to 26%), and the most abundant families were uncultured archaeon from Bathyarchaeota (19% to 30%), Nitrosopumilaceae (12% to 45%) and families within Nanoarchaeaeota (11% to 15%) (Fig. S2C). The most abundant genera were uncultured archaeon from three phyla Bathyarchaeota, Thaumarchaeota and Nanoarchaeaeota (19% to 30%, 12% to 35% and 11% to 15%, respectively).

The most abundant class in DRm and BRmAg included members from the Bathyarchaeota phylum. BRm had Nitrososphaeria and FRm had Thermoplasmata, while in BAg the Marine Benthic Group A and Nitrososphaeria were the dominant classes. The most abundant family in BAg, DRm and BRm was Nitrosopumilaceae, while in BRmAg it was an uncultured bacterium from the phylum Bathyarchaeota and in FRm the highest abundances were of members from the class Thermoplasmata (Fig. S2C).

The weighted Unifrac distance matrix showed no clear separation between the archaeal composition in sediments from the different ecological types of mangroves (Fig. 3B). However, according to the PERMANOVA test (*R*^2^ = 0.453, *p* = 0.001) there is a significant difference in the archaeal composition of ecological types of mangroves. Moreover, the unweighted Unifrac distance matrix shows that the archaea in BAg sediments separates from the other mangrove types (Fig. S6). No physicochemical variables analyzed in this study were related to the differences in archaeal composition between sediments of the different ecological mangrove types (Table S3). There are no archaea indicator taxa from mangroves at Celestún, except for a member of the Nitrosopumilaceae family (Thaumarchaeota phylum) in all ecological types except in FRm, which is relevant regarding their known role as aerobic ammonia oxidizers.

## Discussion

The need to understand all aspects related to the ecology of different mangrove forests and their associated microbes is fundamental to better comprehend their role in global biogeochemical cycles. This study reported a physicochemical gradient observed in sediments, including interstitial salinity, where the FRm mangrove develops in lower salinity due to its proximity to the lagoon, which increased towards the basin mangroves, reaching the highest value in the DRm mangrove, and decreasing again at the furthest point, where the basin mangrove is dominated exclusively by *A. germinans.* Water supply is through runoff from the continental area and groundwater discharges, which favor the variation in interstitial water as well as the nutrient content from each ecological type of mangrove (Twilley & Rivera-Monroy, 2005; Feller et al., 2010). Dwarf-type mangroves, in carbonated areas develop in regions characterized by low concentration of nutrients, mainly phosphorus, which can limit plant growth (Feller et al., 2010). In this study the concentration of nutrients in the sediments of the DRm mangrove was lower compared to the other ecological types of mangroves (Table 3). Although there were certain physicochemical gradients associated to the development of the different ecological types of mangrove here analyzed, the microbial composition was only different for the forest dominated by *A. germinans* (BAg).

Previous work on mangrove sediment bacterial diversity has shown that the most abundant taxa belong to Proteobacteria, Chloroflexi and Bacteroidetes (Andreote et al., 2012; Jiang et al., 2013; Fernandes et al., 2014; Alzubaidy et al., 2016; Zhou et al., 2017; Barreto et al., 2018; Luis et al., 2019) (Table 4), which is similar to the results here presented. Moreover, within Proteobacteria, the class Deltaproteobacteria and Gammaproteobacteria were the most abundant as have been found in other mangroves around the world (Jiang et al., 2013; Fernandes et al., 2014; Alzubaidy et al., 2016; Wu et al., 2016; Ullah et al., 2017; Zhou et al., 2017) (Table 4). Mangrove sediments have been described as mainly anoxic environments with a small oxic zone on the surface, and rich in organic matter (Brodersen et al., 2019). In anaerobic environments, degradation of organic compounds mainly occurs through sulfate reduction mechanisms where sulfate reducing bacteria are involved (Muyzer & Stams, 2008). Phylogenetic lineages recognized to harbor sulfate-reducing bacteria mostly belong to Deltaproteobacteria (Muyzer & Stams, 2008). Interestingly, this was the most abundant class in all sediments from different ecological types of mangrove forests except for the sediments from BAg which showed Gammaproteobacteria as the most abundant class. Within Deltaproteobacteria, the family Desulfobacteraceae showed the highest abundances mainly due to the presence of the Sva0081 sediment group of bacteria which has been reported as an uncultured, possibly microaerophilic bacterium, found in marine sediments (Zheng, Wang & Liu, 2014; Liu et al., 2015; Probandt et al., 2017; Coskun et al., 2019). This microorganism has genes capable of oxidizing H_2_ (Dyksma et al., 2018a), reducing sulfate (Fan et al., 2018) and assimilating acetate in coastal sediments (Dyksma et al., 2018b). *Desulfatiglans* was the second most abundant genus from the Desulfarculaceae in sediments from BAg, DRm and BRmAg and the most abundant in BRm and FRm. Microorganisms from this genus have been found in marine sediments (Jochum et al., 2018) and have been isolated from sediments polluted with phenolic compounds (Jochum et al., 2018). Pollution by aromatic compounds in mangrove sediments has been reported around the world (Tam et al., 2001; Ebrahimi-Sirizi & Riyahi-Bakhtiyari, 2013; Li et al., 2014). Furthermore, there are reports of exposure to toxic compounds by anthropogenic activities in the YP (Arcega-Cabrera et al., 2015) which could explain the high abundances of *Desulfatiglans* as it is capable of degrading aromatic compounds (Xie & Müller, 2018).

**Table 4:** Microbial community composition in sediments from mangroves distributed worldwide.

Mangrove specie	Methodology	Bacterial composition	Archaeal composition	Study site	Reference
*Kandelia obovata Acanthus ilicifolius*	V6 16S rRNA Solexa Genome analyzer (GAII)	Rhizosphere: Nitrospirae, Acidobacteria Bulk sediment: *Proteobacteria* (*Deltaproteobacteria*), *Epsilonproteobacteria*	No data	China	Jiang et al. (2013)
*Avicennia marina*	Metagenomics Pyrosequencing 454 GS FLX Titanium	*Proteobacteria* (*Deltaproteobacteria*: (*Desulfobacterales*, *Desulfuromonadales, Desulfovibrionales, Syntrophobacterales), Gammaproteobacteria (Chromatiales, Alteromonadales, Pseudomonadales, Oceanospirillales, Enterobacteriales), Alphaproteobacteria (Rhizobiales, Rhodobacterales)), Bacteroidetes, Firmicutes*	*Crenarchaeota Euryarchaeota* *Methanosarcinales Thermococcales Methanococcales Methanobacteriales Halobacteriales Nitrosopumilales (Thaumarchaeota)*	Red Sea, Saudi Arabia	Alzubaidy et al. (2016)
*Bruguiera gymnorrhiza* *K. candel* *Aegiceras corniculatum* Rhizosphere	V4-V5 16S rRNA Illumina HiSeq	*Proteobacteria (Deltaproteobacteria, Gammaproteobacteria), Chloroflexi, Bacteroidetes*	*Euryarchaeota Crenarchaeota*	China	Wu et al. (2016)
*A. marina*	V3-V4 16S rRNA Illumina MiSeq	*Proteobacteria (Gammaproteobacteria, Deltaproteobacteria), Actinobacteria,* *Firmicutes*	No data	Red Sea, Saudi Arabia	Ullah et al. (2017)
*Sonneratia caseolaris B. sexángula Rhizophora apiculata Xylocarpus granatum Heritiera littoralis* *B. gymnorrhiza* *R. mucronata* *R. apiculata* *A. marina* *Ceriops tagal* *K. candel* *Nypa fructicans*	V4 16S rRNA Illumina MiSeq	Protected site: *Proteobacteria, Acidobacteria, Actinobacteria* Unprotected site: *Proteobacteria, Chloroflexi and Bacteroidetes*	*Thaumarchaeota Crenarchaeota Euryarchaeota*	Hainan Island, China	Yun, Deng & Zhang (2017)
*Laguncularia racemosa* *Distichlis spicata*	V3-V5 16S rRNA Illumina MiSeq	*Proteobacteria, Chloroflexi, Firmicutes*	No data	Florida	Barreto et al. (2018)
*K. obovata*	V1-V2 16S rRNA Ion Torrent sequencing	*Proteobacteria (Deltaproteobacteria (Desulfuromonadaceae, Desulfobulbaceae), Gammaproteobacteria (Piscirickettsiaceae)* *Bacteroidetes,*	*Bathyarchaeota Euryarchaeota*	Hong Kong	Cheung et al. (2018)
*A. officinalis* *A. alba* *S. alba* *A. illicifolius* *A. corniculatum Exoecaria agallocha*	V4-V3 16S rRNA Illumina	*Proteobacteria (Gammaproteobacteria (Psychrobacter, Halomonas, Pseudomonas), Alphaproteobacteria))* *Firmicutes, Actinobacteria*	*Euryarchaeota (Halobacteria)*	Goa, India	Haldar & Nazareth (2018)
*S. caseolaris* *Sueda fruticosa Urochondra setulosa*	V3-V4 16S rRNA Illumina MiSeq sequencing	*Proteobacteria (Gammaproteobacteria (Marinobacterium), Alphaproteobacteria, Deltaproteobacteria (Desulfobacca, Desulfotalea, Desulfobulbus, Desulfomonile, Desulfovibrio, Desulfosarcina), Bacteroidetes (Flavobacteria, Sphingobacteria), Firmicutes (Bacilli)*	No data	Maharashtra, India	Paingankar & Deobagkar (2018)
*A. corniculatum*	V3-V4 16S rRNA Illumina	*Proteobacteria (Deltaproteobacteria, Gammaproteobacteria), Chloroflexi,* *Bacteroidetes*	No data	China	Lin et al. (2019)
*A. marina* *R. stylosa*	V3-V5 16S rRNA Illumina MiSeq sequencing	*Proteobacteria (Deltaproteobacteria (Desulfobacterales, Syntrophomonadales), Gammaproteobacteria), Chloroflexi (Anaerolinae)*	*Bathyarchaeota Euryarchaeota*	New Caledonian	Luis et al. (2019)

The second most abundant class found in sediments from all mangroves, except BAg, was Gammaproteobacteria mainly represented by Nitrosococcaceae (AqS1). AqS1 is a sulfur-oxidizing microorganism capable of carbon monoxide oxidation and inorganic phosphate assimilation. This bacterium has been reported as a symbiont of the coral reef demosponge *Amphimedon queenslandica* (Gauthier, Watson & Degnan, 2016). Sulfur oxidation is an important step in the biogeochemical cycling of sulfur in anoxic environments with a thin layer of aerobic conditions (SamKamaleson & Gonsalves, 2019), making AqS1 a good candidate to perform this function within the mangrove sediments. Archaea have been suggested as relevant players in carbon, nitrogen and sulfur cycles in mangroves (Zhang et al., 2018). Archaea in Celestún sediments were represented primarily by Thaumarchaeota (Nitrososphaeria). Cultivated microorganisms within this phylum have been isolated from soil, marine, estuarine environments and wastewater treatment plants. They are chemolithoautotrophs and are capable of aerobic ammonium oxidation (ammonia-oxidizing archaea) (Kerou, Eloy Alves & Schleper, 2016) and ammonia-oxidation (nitrification) in hypoxic zones (Campbell et al., 2019). Bathyarchaeota was the second most abundant phylum found. This phylum has been reported to fix CO_2_ *via* acetogenesis (He et al., 2016) and is capable of dissimilatory nitrite reduction to ammonium (Lazar et al., 2017). Moreover, metagenomic reconstruction of Bathyarchaeota has suggested their capability for methane production using methylated compounds (Evans et al., 2015) and some studies have proposed their potential role in anaerobic methane oxidation using alternative electron acceptors (Saxton et al., 2016; Valenzuela et al., 2017). Since we were not able to identify methanogenic/methanotrophic microorganisms related to Euryarchaeota at Class level, we hypothesize that Bathyarchaeota could play a fundamental role in the methane cycle in these mangrove ecosystems.

Indicator Taxa Analysis (ITA) can be used to understand the presence of an organism in a specific environment, according to their preferences of niche conditions. These values are independent of the relative abundances of other microorganisms (Dufrêne & Legendre, 1997; De Cáceres et al., 2010). Indicator taxa have been used to identify microorganisms associated with a specific environmental variable or niche, such as agricultural practices (Jiménez-Bueno et al., 2016), gut microbiome (Youngblut et al., 2019), anemone microbiome (Morelan et al., 2019) and soil microbiome (Naylor et al., 2020). Thus, to explore unique associations between sediment bacteria and different mangrove ecotypes, bacterial indicator taxa were determined. The contrast between mangrove forests sediment microbial composition dominated by *A. germinans* and *R. mangle* is very interesting. BAg had the highest number and diversity of indicator taxa while in contrast, sediments from the FRm and BRm mangroves showed few or none indicator taxa, suggesting a cosmopolitan composition for sediment microbes associated with *R. mangle* and a high specificity associated to *A .germinans* mangrove forests in Celestún.

Indicator taxa found in BAg are either strictly anaerobic chemoheterotrophs, such as Anaerolineales (Chloroflexi) (Yamada & Sekiguchi, 2015), or facultative anaerobic microorganisms such, as Ignavibacteriales (Bacteroidetes) (Lino, 2014). Their presence in mangrove sediments suggests degradation of organic matter by electron acceptors other than oxygen. These anoxic conditions could also be formed during flooding periods that inhibit direct exchange of gases with the atmosphere. However, sediments from BAg mangroves show *Phaeodactylibacter,* a strict aerobic and chemoheterotrophic microorganism as indicator taxa, suggesting that these sediments are still in contact with oxygenated waters (Chen et al., 2014; Lei et al., 2015).

Moreover, obligate halophilic microorganisms from Deltaproteobacteria, such as *Haliangium* Garcia & Müller, 2014 or SAR324, a heterotrophic microorganism found in the Red Sea (Haroon, Thompson & Stingl, 2016), were found as indicator taxa in mangrove sediments from Celestún (BRmAg and FRm respectively), probably due to their high salinity. In addition to this, some indicator taxa from BAg participate in the nitrogen cycle in marine environments including Nitrosomonadaceae which are lithoautotrophic ammonia oxidizers (Prosser, Head & Stein, 2014), *Calothrix* (which is a nitrogen fixer) and *Phaeodactylibacter* that reduces nitrate (Kompantseva et al., 2017; Chen et al., 2014; Lei et al., 2015). The most abundant bacterial indicator taxa in the BAg sediments belonged to the order MBMPE27. Members of this order are exclusively uncultured Gammaproteobacteria reported in environments such as moss pillars, hot springs, hydrogenetic ferromanganese crusts, massive sulfide deposits, deep sea sediments, coral reefs and other aquatic environments (Kimura et al., 2006; Santelli et al., 2008; Reis et al., 2009; Schauer et al., 2010; Nitahara et al., 2011; Nakai et al., 2012; Kato et al., 2015). Little is known about these microorganisms, as no strain has been isolated and there are no records of an assembled genome. However, their wide distribution in diverse marine environments suggests they have an important role in nutrient cycling.

Proteobacteria, Calditrichaeota, Chloroflexi, Spirochaetes, Nitrospinae and Zixibacteria are indicator taxa shared between spatially closer mangroves, all dominated by *R. mangle* (DRm, BRm, BRmAg and FRm). Within these indicator taxa there are microorganisms that reduce sulfate and/or sulfur including Desulfobulbaceae, Syntrophobacteraceae and Desulfobacteraceae (Kuever, 2014a; Kuever, 2014b; Laban et al., 2015), which, again, highlights the important role of these microorganisms in degrading organic matter through sulfate reduction mechanisms. Furthermore, the Desulfobulbaceae includes microorganisms able to degrade hydrocarbons under anaerobic conditions (Laban et al., 2015), as well as *Dehalococcoidia*, a strict anaerobe member of Chloroflexi capable of organohalide respiration, a process that has applications in bioremediation of chlorinated compounds (Löffler et al., 2013; Biderre-Petit et al., 2016).

## Conclusions

This study represents the first characterization of sediment bacterial and archaeal diversity in different ecological types of mangroves in Celestún, a representative site for mangrove research in the Yucatan Peninsula. All ecological types of mangroves, except for FRm, had Nitrosopumilaceae as their indicator taxa. Members of this family are capable of aerobic ammonium oxidation, an important process that regulates the level of nitrogen in marine ecosystems, however the mechanisms by which this process is accomplished, are not completely understood (Taylor & Kurtz Jr, 2020). Nevertheless, their high abundances in these mangroves highlights the importance of nitrogen cycling. Finally, all mangrove sediments from Celestún harbor microorganisms that have been reported as capable of participating in organic matter, nitrogen and sulfur cycling, highlighting the role of the microbial component in these ecosystems. Microorganisms that have the ability to degrade toxic compounds, to which mangroves are exposed due to anthropogenic activities, were present in all ecological mangrove types.

The microbial composition associated to basin mangroves dominated by *A. germinans* is significantly different from the other ecological types, suggesting a particular microbiome, whereas all mangrove types formed by *R. mangle*, had a generalist microbial component associated to their root sediments. Perhaps these differences in microbial composition will help future restoration efforts with *A. germinans*, which appears to have a specific sediment microbiome, which despite having the lowest microbial diversity, had the greatest number of indicator species.

##  Supplemental Information

10.7717/peerj.14587/supp-1 Supplemental Information 1 Supplemental Figures and Tables

10.7717/peerj.14587/supp-2 Supplemental Information 2 Physicochemical characteristics from mangrove sediment sampled in 2018 raw data Data from basin, dwarf and fringe mangroves from Celestún, Yucatán, Mexico

## Additional Information and Declarations

### DNA Deposition

The following information was supplied regarding the deposition of DNA sequences: Sequence reads are available at the NCBI Sequence Read Archive Bioproject and SRA: PRJNA550111, SRR9973295–SRR9973347.

### Data Availability

The following information was supplied regarding data availability: The raw data is available as a Supplemental File.
