# Supplementary material for: Sediment microbial community structure associated to different ecological types of mangroves in Celestún, a coastal lagoon in the Yucatan Peninsula, Mexico
Source: PeerJ. 2023 Feb 8;11:e14587. doi: 10.7717/peerj.14587 (PMC9921989; doi:10.7717/peerj.14587)
Supplement: Supplemental Information 1 [file peerj-11-14587-s001.docx]

Supplementary material

Supplementary Table S1. Diversity index for sediment microbial diversity (*16SrRNA* gene,V4 region) of different ecological mangrove forests in Celestún, Yucatán

|  | Bacteria | | | Archaea | | |
| --- | --- | --- | --- | --- | --- | --- |
| Mangrove | Simpson | Shannon | Observed ASVs | Simpson | Shannon | Observed ASVs |
| BAg | 0.99 ± 0.003 | 8.00 ± 0.245 | 597.92 ± 89.14 | 0.83 ± 0.07 | 3.81 ± 0.46 | 39.63 ± 5.27 |
| DRm | 1.00 ± 0.001 | 9.40 ± 0.678 | 1320.73 ± 373.53 | 0.91 ± 0.07 | 5.45 ± 0.79 | 139.90 ± 36.68 |
| BRm | 1.00 ± 0.002 | 9.30 ± 0.361 | 1510.38 ± 376.42 | 0.93 ± 0.05 | 5.65 ± 0.61 | 154.50 ± 40.79 |
| BRmAg | 1.00 ± 0.001 | 9.60 ± 0.296 | 1597.64 ± 329.69 | 0.97 ± 0.01 | 6.18 ± 0.43 | 163.00 ± 41.29 |
| FRm | 1.00 ± 0.001 | 9.60 ± 0.388 | 1513.08 ± 376.16 | 0.96 ± 0.04 | 6.53 ± 0.88 | 193.08 ± 48.72 |
| Kruskal-Wallis, *p-value* | 5.406^-7^ | 2.928^-6^ | 9.481^-6^ | 9.085^-5^ | 1.135^-5^ | 5.908^-5^ |

Post hoc Dunn test: **Bacteria** *p-value* <0.001 (Shannon: BAg vs FRm, BAg vs BRmAg; Simpson: BAg vs FRm, BAg vs BRmAg; Observed_otus: BAg vs FRm, BAg vs BRmAg, BAg vs BRm, BAg vs DRm). **Archaea** *p-value* <0.001 (Shannon: BAg vs FRm, BAg vs BRmAg; Simpson: BAg vs FRm; BAg vs BRmAg; Observe_otus: BAg vs FRm, BAg vs BRmAg).

Supplementary Table S2. Bacterial and Archaeal phyla showing significant differences in composition between ecological types of mangrove forests in Celestún, Yucatán (Kruskal-Wallis p < 0.001).

| Domain | Phyla | Pos hoc Dunn test *p-value* | | | | | |
| --- | --- | --- | --- | --- | --- | --- | --- |
|  |  | BAg vs FRm | BAg vs BRm | BAg vs BRmAg | BRmAg vs BRm | BRmAg vs FRm | BRm vs FRm |
| Bacteria | Proteobacteria | 0.787 | 1.000 | < 0.050 | < 0.010 | < 0.001 | 1.000 |
|  | Chloroflexi | 0.010 | 1.000 | 0.228 | < 0.100 | < 0.001 | 0.174 |
|  | Gemmatimonadetes | < 0.001 | 1.000 | 0.178 | 0.429 | 0.157 | < 0.001 |
|  | Acidobacteria | < 0.010 | < 0.050 | < 0.010 | 1.000 | < 0.050 | < 0.010 |
|  | Nitrospirae | < 0.010 | < 0.001 | 0.368 | < 0.100 | 1.000 | 1.000 |
|  | Firmicutes | < 0.001 | < 0.010 | < 0.050 | 1.000 | 1.000 | 1.000 |
| Archaea | Crenarchaeota | < 0.001 | 0.177 | 1.000 | 1.000 | < 0.010 | 1.000 |
|  | Thaumarchaeota | < 0.001 | 0.300 | < 0.010 | 1.000 | 1.000 | 0.746 |
|  | Euryarchaeota | < 0.001 | 0.389 | < 0.001 | 0.488 | 1.000 | 0.151 |

Supplementary Table 3. PERMANOVA test performed on the Weighted Unifrac distance matrix and the significantly different physicochemical characteristics from different ecological types of mangrove forests in Celestún, Yucatán.

| Variable | Bacteria | | Archaea | |
| --- | --- | --- | --- | --- |
|  | P | R^2^ | P | R^2^ |
| **Ecological type** | 0.001 | 0.468 | 0.001 | 0.453 |
| Interstitial water salinity | 0.654 | 0.028 | 0.393 | 0.011 |
| Interstitial water temperature | 0.562 | 0.008 | 0.395 | 0.014 |
| Superficial water salinity | 0.951 | 0.004 | 0.423 | 0.010 |

Supplementary Table 4. IndVal values of bacterial indicator taxa from ecological types of mangrove forests from Celestún, Yucatán (p < 0.001).

| Mangrove type | Taxa | IndVal |
| --- | --- | --- |
| BAg | Gammaproteobacteria;JTB23 | 1.000 |
|  | Gammaproteobacteria;MBMPE27 | 0.976 |
|  | Gammaproteobacteria;MBMPE27;uncultured bacterium | 0.996 |
|  | Deltaproteobacteria;Syntrophobacterales;Syntrophobacteraceae;uncultured bacterium | 0.989 |
|  | Alphaproteobacteria;uncultured bacterium | 0.965 |
|  | Gammaproteobacteria;Betaproteobacteriales;Nitrosomonadaceae | 0.957 |
|  | Deltaproteobacteria;NB1-j;uncultured bacterium | 0.954 |
|  | Gammaproteobacteria; other | 0.96 |
|  | Gemmatimonadetes;Gemmatimonadales;Gemmatimonadaceae;uncultured bacterium | 0.951 |
|  | Gemmatimonadetes;Gemmatimonadales;Gemmatimonadaceae;uncultured bacterium | 0.957 |
|  | Gemmatimonadetes;Gemmatimonadales;Gemmatimonadaceae;uncultured bacterium | 0.957 |
|  | Gemmatimonadetes;Gemmatimonadales;Gemmatimonadaceae;uncultured bacterium | 0.952 |
|  | Anaerolineae;Anaerolineales;Anaerolineaceae;uncultured bacterium | 0.992 |
|  | Anaerolineae;Anaerolineales;Anaerolineaceae;uncultured bacterium | 0.957 |
|  | Subgroup 21;uncultured bacterium | 0.992 |
|  | Calditrichia;Calditrichales;Calditrichaceae;Calorithrix | 1.000 |
|  | Ignavibacteria;Ignavibacteriales | 0.957 |
|  | Bacteroidia;Chitinophagales;Saprospiraceae;Phaeodactylibacter | 0.98 |
|  | Thermodesulfovibrionia;uncultured bacterium | 1.000 |
|  | Planctomycetes;Phycisphaerae | 0.957 |
| DRm | Deltaproteobacteria;NB1-j;uncultured bacterium | 1.000 |
|  | Gammaproteobacteria;MBMPE27 | 1.000 |
|  | Phycisphaerae;Phycisphaerales;Phycisphaeraceae;SM1A02;uncultured bacterium | 1.000 |
|  | Gammaproteobacteria;EPR3968-O8a-Bc78 | 0.993 |
|  | Gammaproteobacteria; other | 0.979 |
| BRmAg | Alphaproteobacteria;Rhodovibrionales;Kiloniellaceae | 1.000 |
|  | Deltaproteobacteria;Bdellovibrionales;Bdellovibrionaceae;Bdellovibrio;uncultured bacterium | 1.000 |
|  | Deltaproteobacteria;NB1-j;uncultured bacterium | 0.989 |
|  | Deltaproteobacteria;NB1-j;uncultured bacterium | 0.988 |
|  | Deltaproteobacteria;Myxococcales;Sandaracinaceae;Sandaracinus | 0.987 |
|  | Gammaproteobacteria;Xanthomonadales;Rhodanobacteraceae;Luteibacter | 0.986 |
|  | Gammaproteobacteria;Steroidobacterales;Woeseiaceae;Woeseia | 0.978 |
|  | Deltaproteobacteria;Desulfobacterales;Desulfobacteraceae;Sva0081 sediment group | 0.955 |
|  | Gammaproteobacteria;Nitrosococcales;Nitrosococcaceae;AqS1;uncultured bacterium | 0.954 |
|  | Planctomycetacia;Planctomycetales;uncultured bacterium | 0.953 |
|  | Gammaproteobacteria | 0.953 |
|  | Deltaproteobacteria;Myxococcales;Haliangiaceae;Haliangium;uncultured bacterium | 0.953 |
|  | Chlorobia;Chlorobiales;Chlorobiaceae;Chlorobium | 0.953 |
|  | Gammaproteobacteria;B2M28;uncultured bacterium | 0.953 |
|  | Bacteroidia;Bacteroidales;Bacteroidetes BD2-2 | 0.953 |
|  | Deltaproteobacteria;Myxococcales;Sandaracinaceae;uncultured delta proteobacterium | 0.953 |
| FRm | Deltaproteobacteria;SAR324 clade(Marine group B) | 0.957 |
|  | Deltaproteobacteria;SAR324 clade(Marine group B) | 0.956 |

Supplementary Table S5. Pairwise Permanova results.

|  | Bacteria | | | Archaea | |
| --- | --- | --- | --- | --- | --- |
| Group 1 | Group 2 | pseudo-F | p-value | pseudo-F | p-value |
| BAg | DRm | 12.253 | 0.001 | 12.827 | 0.001 |
|  | BRm | 20.548 | 0.001 | 28.331 | 0.001 |
|  | FRm | 16.765 | 0.001 | 21.322 | 0.001 |
|  | BRmAg | 13.902 | 0.001 | 12.400 | 0.001 |
| DRm | BRm | 6.366 | 0.001 | 2.513 | 0.070 |
|  | FRm | 7.416 | 0.001 | 4.766 | 0.017 |
|  | BRmAg | 4.856 | 0.001 | 2.931 | 0.021 |
| BRm | FRm | 10.221 | 0.001 | 3.221 | 0.031 |
|  | BRmAg | 6.274 | 0.001 | 5.441 | 0.003 |
| FRm | BRmAg | 6.090 | 0.003 | 7.163 | 0.003 |


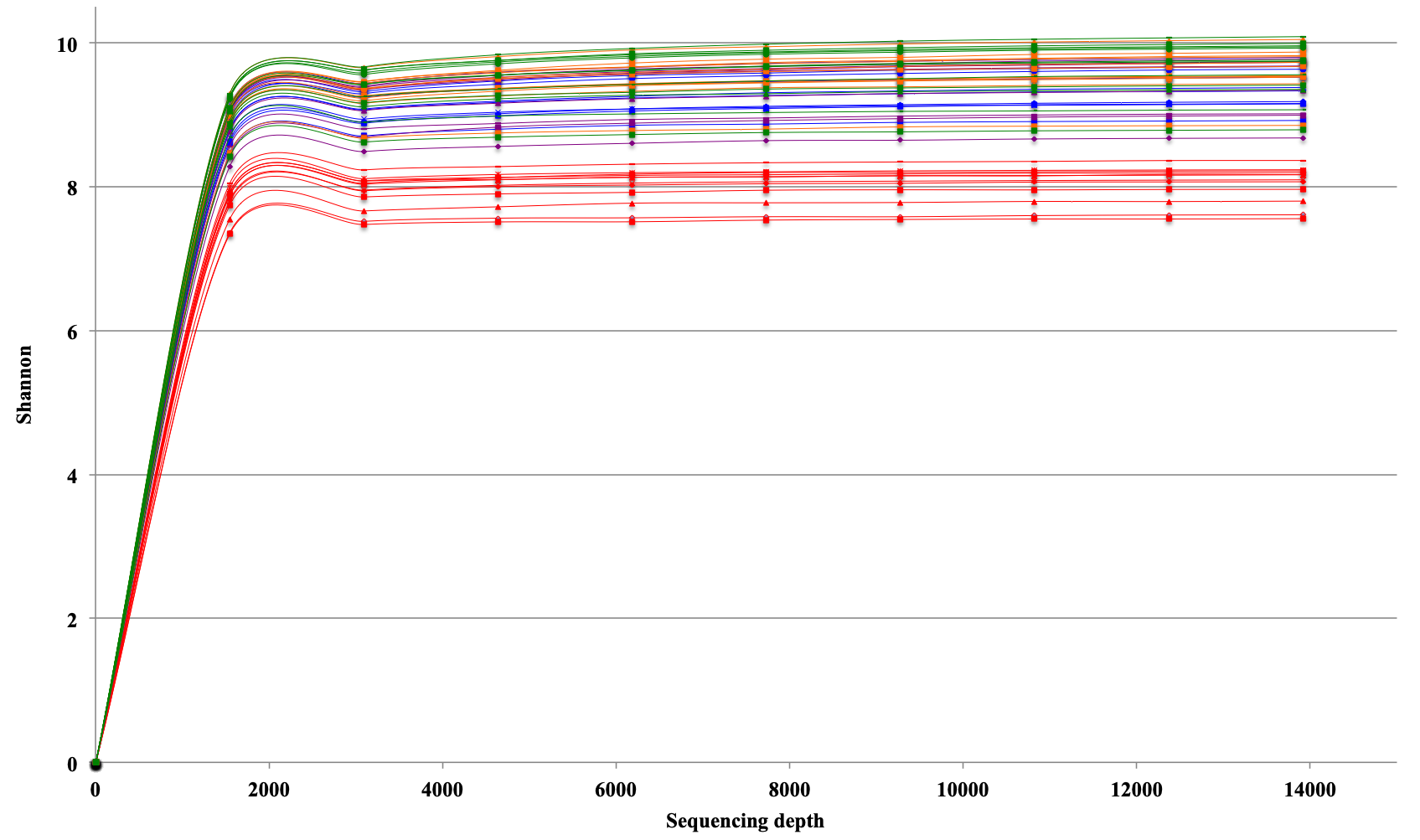


Figure S1. Rarefaction curves, based on Shannon index, for 16SrDNA-V4 sequences assigned to Bacteria in different ecological mangrove forests: BAg (**

**), DRm (**

**), BRm (**

**), BRmAg (**

**), FRm (**

**)


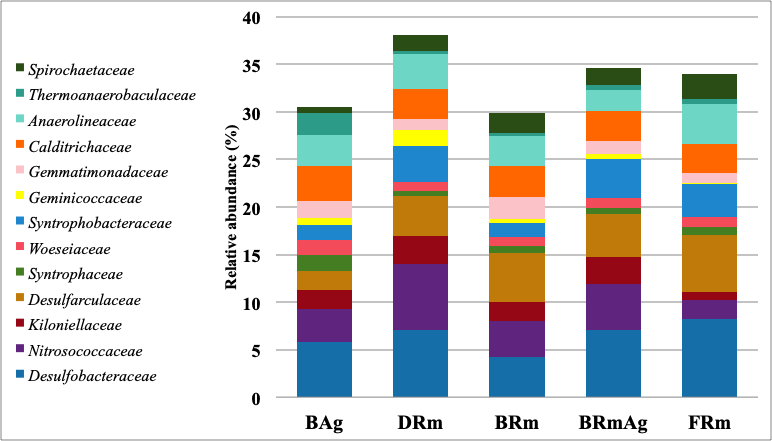


A.


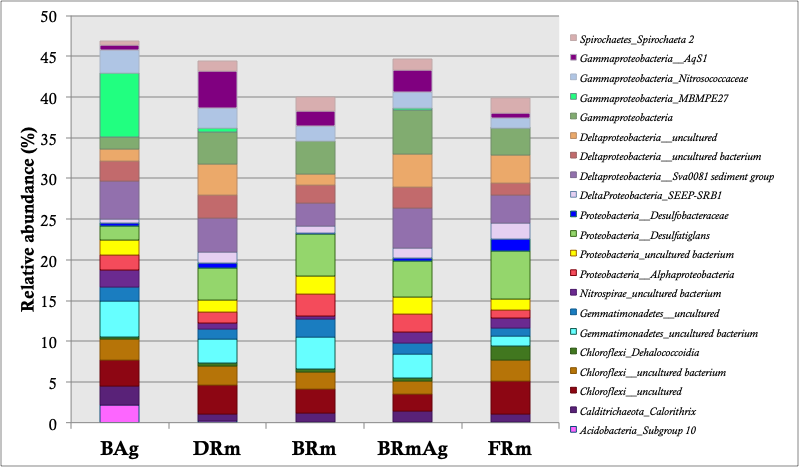


B.


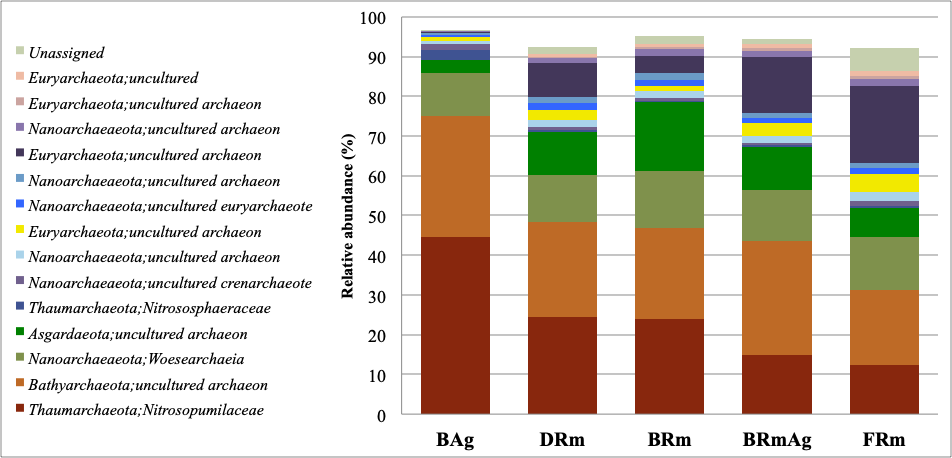


C.

Figure S2. Most abundant bacterial taxa in ecological mangrove forests in Celestún, Yucatán: BAg, DRm, BRm, BRmAg, FRm. A) bacterial family and B) genus; C) Archaea.


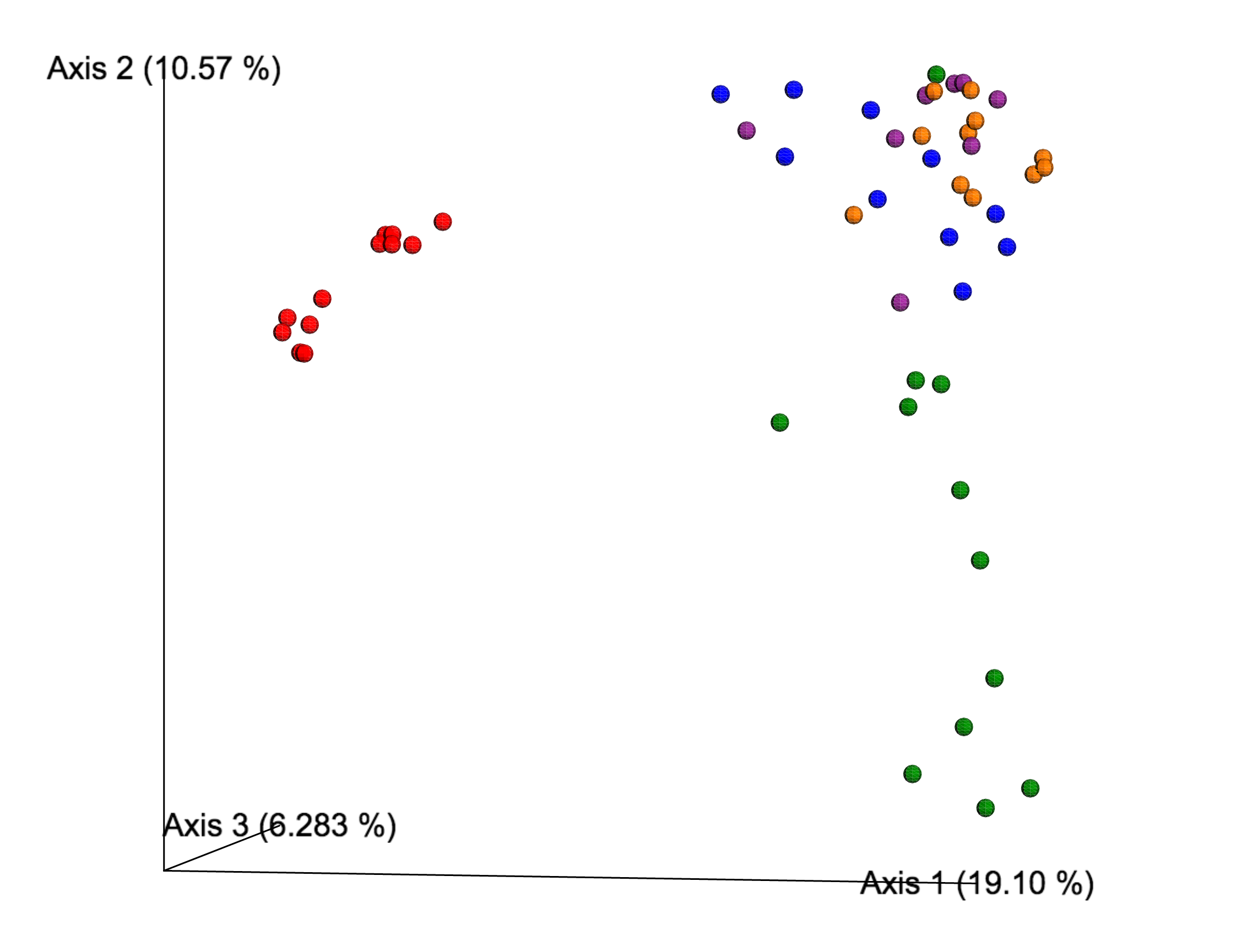


Figure S3. Unweighted Unifrac of bacterial 16SrRNA-V4 sequences of sediments from different ecological mangrove forests in Celestún, Yucatán: BAg (**

**), DRm (**

**), BRm (**

**), BRmAg (**

**), FRm (**

**).


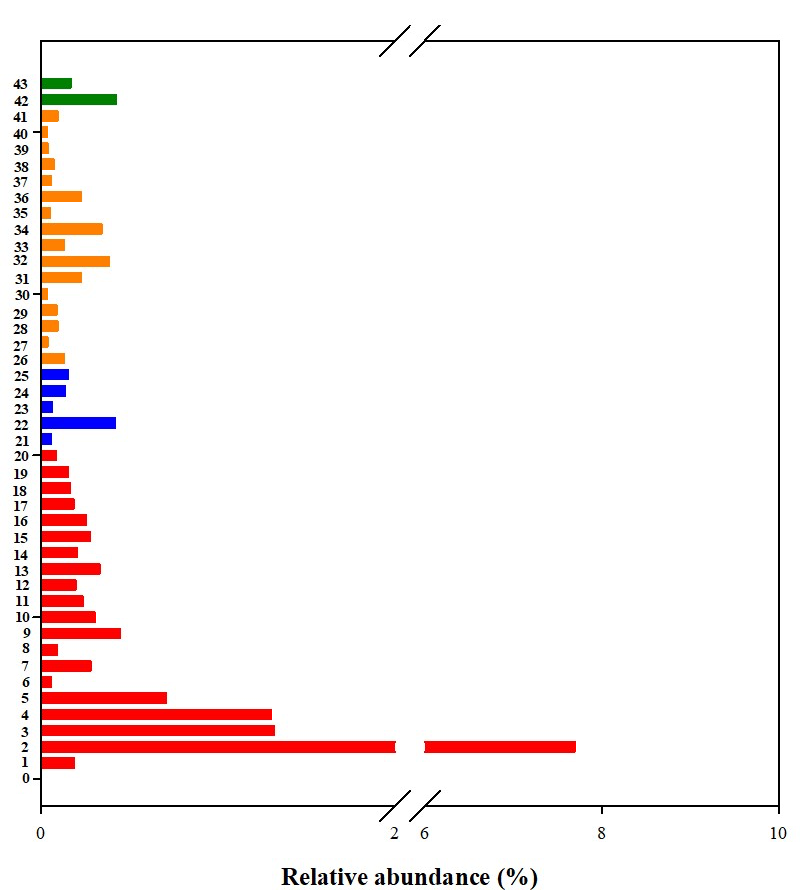


Figure S4. Relative abundance of bacterial indicator taxa of different ecological types of mangrove forests from Celestún: BAg (**

**), DRm (**

**), BRmAg (**

**), FRm (**

**). BRm does not have any bacterial indicator taxa. Proteobacteria (Gammaproteobacteria): ^1^JTB23, ^2,3,22^MBMPE27, ^6^Nitrosomonadaceae, ^25^EPR3968-O8a-Bc78, ^31^Luteibacter, ^32^Woeseia, ^34^AqS1, ^39^B2M28; Proteobacteria (Deltaproteobacteria): ^4^Syntrophobacteraceae, ^7,21,28,29^NB1-j, *^27^Bdellovibrio*, ^30^Sandaracinus, ^33^Sva0081 sediment group, ^37^Haliangium, ^41^Sandaracinaceae, ^42,43^SAR324 clade, Proteobacteria (Alphaproteobacteria): ^26^Kiloniellaceae, Gemmatimonadetes: ^9,10,11,12^Gemmatimonadaceae; Chloroflexi: ^13,14^Anaerolineaceae, ^15^Acidobacteria, Calditrichaeota: ^16^Calorithrix; Bacteroidetes: ^17^Ignavibacteriales, ^18^Phaeodactylibacter, ^38^Chlorobium, ^40^Bacteroidales; Nitrospirae: ^19^Thermodesulfovibrionia, Planctomycetes: ^20^Phycisphaerae, ^23^Phycisphaeraceae, ^35^Planctomycetacia.


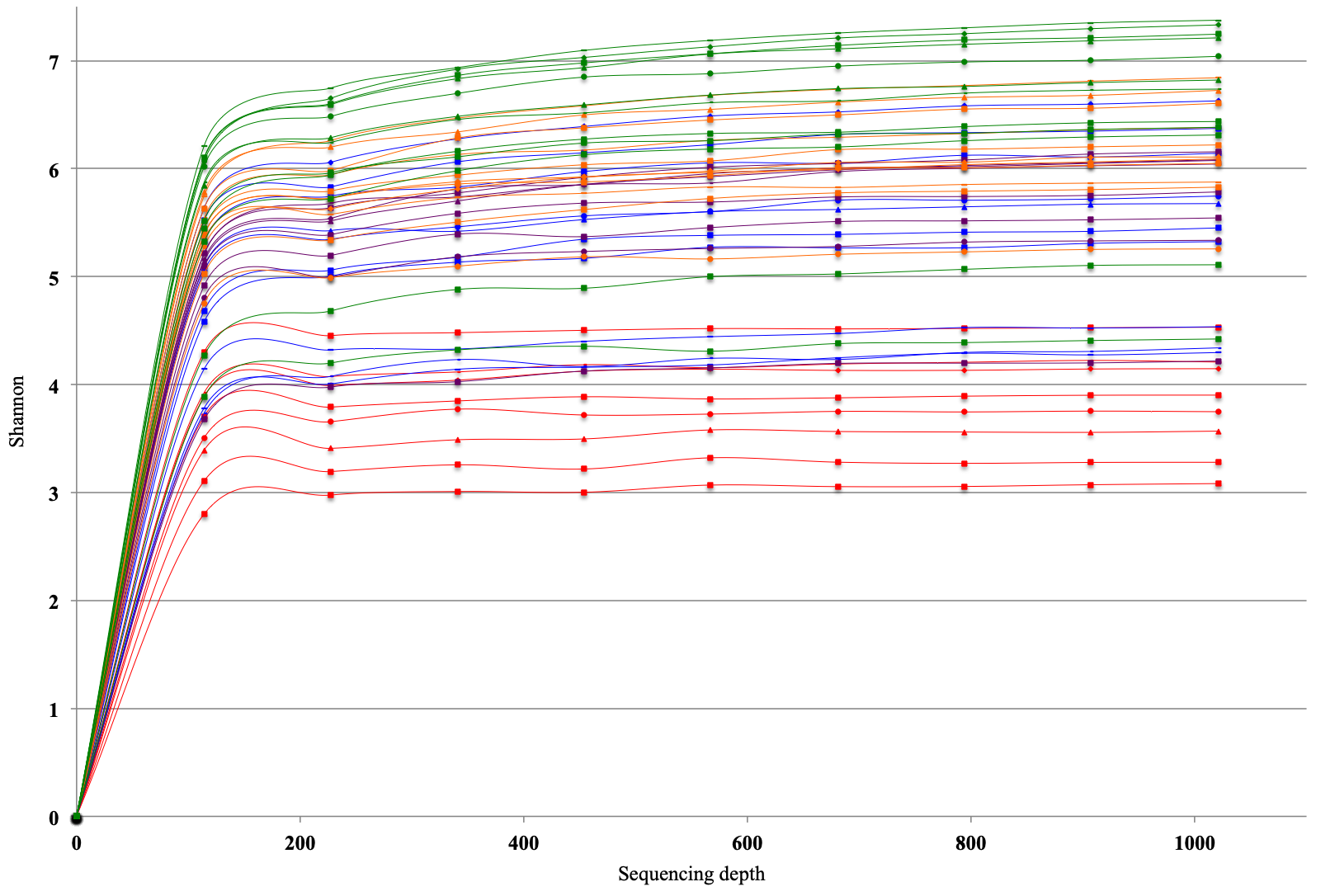
Figure S5. Rarefaction curves for 16SrRNA-V4 sequences of Archaea, based on Shannon index, for different ecological mangrove forests: BAg(**

**), DRm(**

**), BRm (**

**), BRmAg (**

**), FRm (**

**).


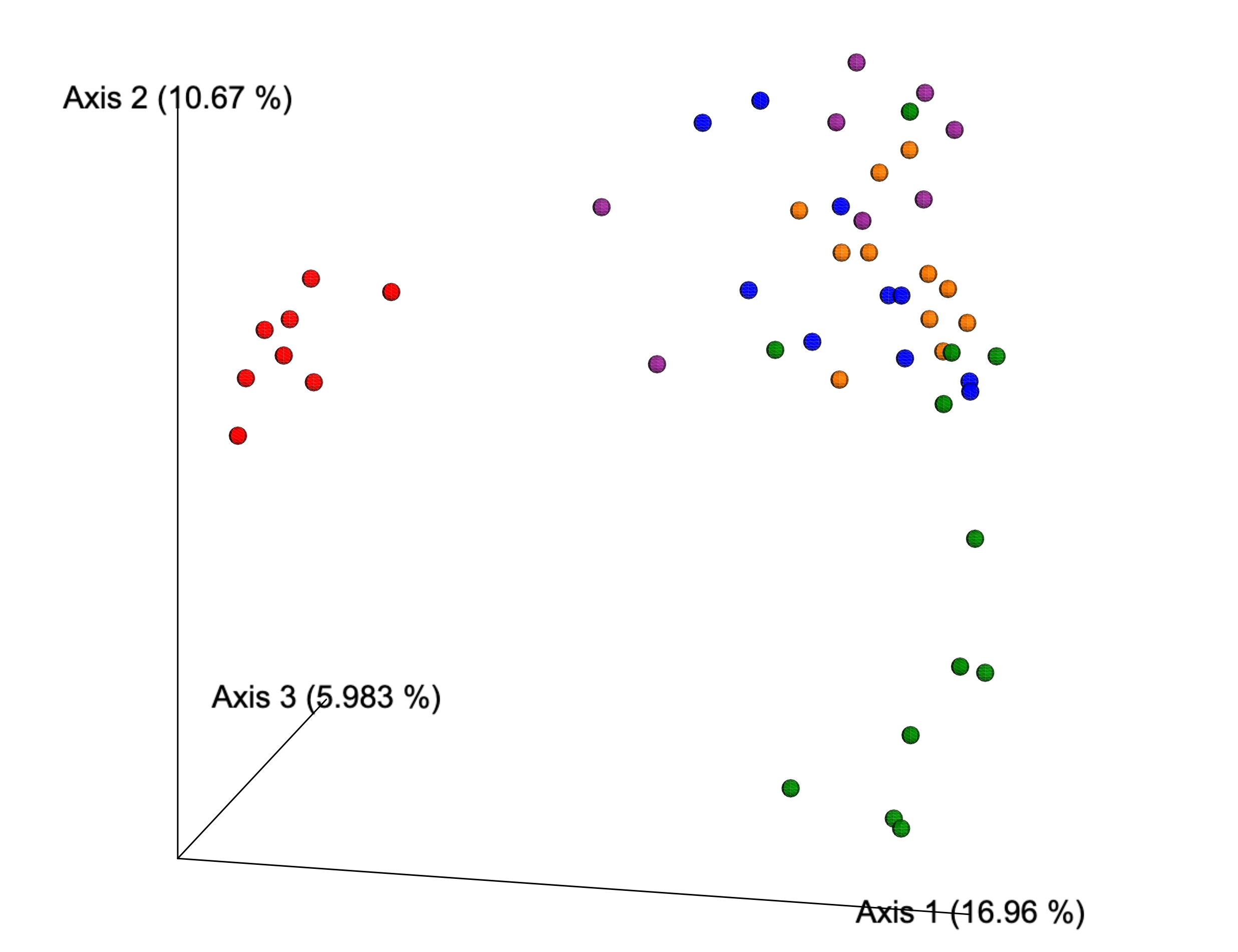


Figure S6. Unweighted Unifrac of archeal 16SrRNA-V4 sequences of sediments from different ecological mangrove forests in Celestún, Yucatán: BAg (**

**), DRm (**

**),BRm (**

**), BRmAg (**

**), FRm (**

**).
